# High-Throughput Determination of Infectious Virus Titers by Kinetic Measurement of Infection-Induced Changes in Cell Morphology

**DOI:** 10.3390/ijms25158076

**Published:** 2024-07-24

**Authors:** Dominik Hotter, Marco Kunzelmann, Franziska Kiefer, Chiara Leukhardt, Carolin Fackler, Stefan Jäger, Johannes Solzin

**Affiliations:** 1 Boehringer Ingelheim Pharma GmbH & Co. KG, Viral Therapeutics Center, 88397 Biberach an der Riss, Germany; 2 Boehringer Ingelheim Pharma GmbH & Co. KG, Development Biologicals, 88397 Biberach an der Riss, Germany; 3 Boehringer Ingelheim Pharma GmbH & Co. KG, Central Nervous System Diseases Research, 88397 Biberach an der Riss, Germany

**Keywords:** infectious virus titer, kinetic imaging, cell rounding, infectivity assay, TCID50, ATMP, virus-based therapeutics, vaccine, high throughput

## Abstract

Infectivity assays are the key analytical technology for the development and manufacturing of virus-based therapeutics. Here, we introduce a novel assay format that utilizes label-free bright-field images to determine the kinetics of infection-dependent changes in cell morphology. In particular, cell rounding is directly proportional to the amount of infectious virus applied, enabling rapid determination of viral titers in relation to a standard curve. Our kinetic infectious virus titer (KIT) assay is stability-indicating and, due to its sensitive readout method, provides results within 24 h post-infection. Compared to traditional infectivity assays, which depend on a single readout of an infection endpoint, cumulated analysis of kinetic data by a fit model results in precise results (CV < 20%) based on only three wells per sample. This approach allows for a high throughput with ~400 samples processed by a single operator per week. We demonstrate the applicability of the KIT assay for the genetically engineered oncolytic VSV-GP, Newcastle disease virus (NDV), and parapoxvirus ovis (ORFV), but it can potentially be extended to a wide range of viruses that induce morphological changes upon infection. The versatility of this assay, combined with its independence from specific instruments or software, makes it a promising solution to overcome the analytical bottleneck in infectivity assays within the pharmaceutical industry and as a routine method in academic research.

## 1. Introduction

Engineered viruses have great potential for therapeutic applications, which is reflected by the increasing number of clinical trials and the approval of virus-based therapeutics in various clinical indications including cancer, infectious diseases, and gene therapy [1]. The high structural and biological complexity of these advanced therapeutic medicinal products (ATMPs) represents a challenge for pharmaceutical development, production, and quality control. In most cases, the infectivity of virus-based therapeutics is of crucial importance as it is the critical quality attribute that reflects viral potency and often represents the parameter defining the dose administered to the patient. A fast, precise, and accurate method to measure infectious virus titers is therefore one of the key requirements to successfully develop and manufacture such virus-based drugs in a cost-effective manner and finally to ensure patients’ safety. Physico-chemical analytical methods such as nanoparticle tracking analysis (NTA), microfluidic resistive pulse sensing (MRPS), enzyme-linked immunosorbent assay (ELISA), or PCR-based methods are usually fast and easy to apply and can be used to determine concentrations of viral particles or genomic titers [2]. These types of assays, however, cannot measure viral infectivity as they do not reflect viral functionality and fitness in the complex interplay that allows the virus to enter a target cell, replicate, and spread its progeny. The use of cell-based assays is therefore the gold standard to determine infectious virus titers.

The plaque assay and the TCID50 assay represent two frequently used examples of such traditional cell-based infectivity assays. In the plaque assay, adherent cells are infected with different concentrations of the virus sample before adding a viscous overlay medium, which limits virus spread to directly neighboring cells. This results in localized infection clusters, originating from a single infection event. These so-called plaques, which are identified by the presence of cytopathic effects (CPEs) or by virus-specific staining, are then counted to determine the infectious sample titer [3]. Although the TCID50 assay is performed on adherent target cells as well, it follows a different assay principle. The TCID50 assay is an endpoint dilution assay in which serial dilutions of the virus sample are added to the culture wells in multiple replicates. The term endpoint dilution refers to the point within the serial sample dilution at which only a single (or few) or no infectious particles are left. After sufficient time, during which viral replication and spread through the well occur, the presence or absence of virus is evaluated, often by microscopic assessment of infection-induced CPEs [3]. This reduced binary information is then used in the statistical models of Spearman/Kärber or Reed/Münch [4,5,6] to determine the TCID50 (tissue culture infectious dose 50%), describing the sample dilution factor at which half of the inoculated wells are infected. 

The benefits of these traditional infectivity assays are that they are easy to perform, do not require specialized technological equipment, and can be adapted to a large variety of virus–cell combinations. Low precision is, however, a common issue, and depending on the tested virus and readout method, variability of ±0.5 log_10_ is expected [7,8]. The low precision can often be compensated for by combining results from multiple sample dilutions or independent assay repetitions, which comes at the cost of increased hands-on time. Furthermore, the long incubation times required for observing the endpoint make the TCID50 assay low-throughput. Despite the described limitations, the traditional infectivity assays have been used for decades. However, due to the growing complexity and sample size associated with the development of virus-based ATMPs, there is an increasing need for improved concepts for infectivity assays. 

During the execution of traditional infectivity assays, a large part of the hands-on time is spent on the process of plaque counting or microscopic evaluation of CPEs. This is not only a time-consuming procedure but also a source of variability due to subjective operator-dependent judgements. We have previously shown that the implementation of benchtop pipetting robots in combination with an automated procedure for the acquisition and analysis of label-free bright-field images results in a highly standardized and ~4× faster TCID50 assay [9]. Similarly, it was reported that image-based automated counting of plaques increases the robustness and speed of plaque assays [10]. Furthermore, novel technologies offer alternative concepts for the identification and characterization of infected cells. Laser force cytology, for example, uses the principle of optical tweezers combined with microfluidics to analyze changes in the biophysical properties of infected cells. This allows one to correlate different infection metrics with the applied virus dose and to calculate sample titers relative to a calibration curve [11,12]. Dodkins and colleagues applied convolutional neural networks (CNNs), in a machine learning approach, to quantify cellular phenotypes specific to viral infection in label-free bright-field images. This AI-based image processing allowed the precise determination of infectious virus titers for a variety of viruses from different families. At the same time, using subtle early cellular changes as a readout for infected cells significantly shortened the total assay duration, which represents a bottleneck in traditional infectivity assays [13]. The need for measurements at a single late assay endpoint is also alleviated by the xCELLigence Real-Time Cell Analysis (RTCA) system. Kinetically recorded measurements of impedance, which correlates with the integrity of a cell monolayer in multi-well plates with embedded micro-electronic biosensors, enable tracking of virus-induced cell lysis over time. It has been shown that this time-dependent information can be used to quantify infectious Hepatitis A virus (HAV), while reducing the overall assay duration by more than 50% compared to traditional infectivity assays [14].

Our goal was to combine the respective advantages of these previous advances in a fast infectivity assay with high precision and sample throughput, while avoiding weaknesses such as complex data analysis or dependence on proprietary technology provided by a single manufacturer. For our assay development activities, we used VSV-GP, a virus from our oncolytic therapy platform [15,16,17]. We show that infection of BHK-21 cells with VSV-GP induces cell rounding in a time- and dose-dependent manner. The proportion of rounded cells in kinetically acquired bright-field images can be used to determine infectious virus titers relative to a reference standard curve. The resulting assay yields high precision (CV < 20%), shortens the time to results to only 24 h, and increases the sample throughput ~10-fold compared to traditional TCID50 assays. Importantly, the principle of the kinetic infectious virus titer (KIT) assay can be applied to different virus–cell combinations and could therefore be of interest for academic virology and pharmaceutical industry.

## 2. Results

### 2.1. Cell Rounding Occurs Early after Infection and Correlates with the Applied Virus Dose

**Figure 1:**
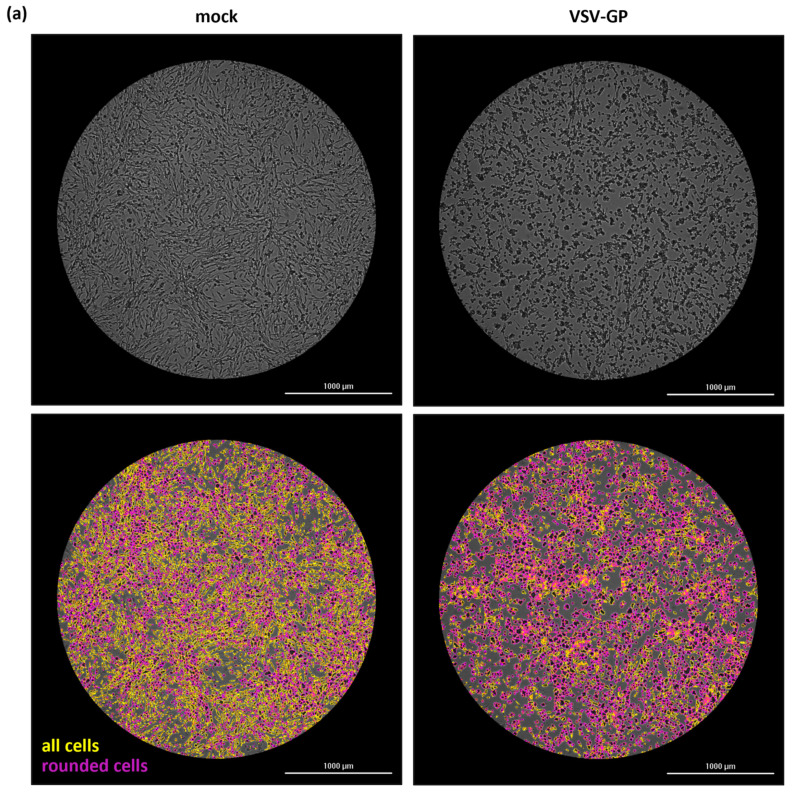

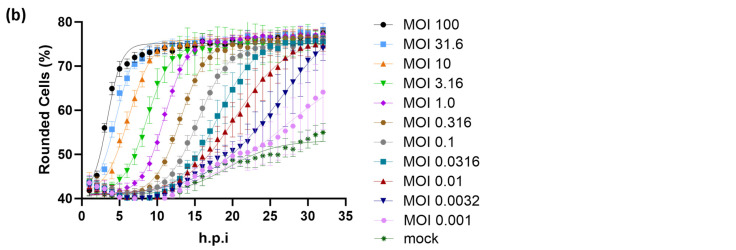
Infection-induced cell rounding in BHK-21 cells. (**a**) Upper panel: Representative bright-field images of mock or VSV-GP-infected (MOI 1) BHK-21 cells, acquired at 10 h post-infection (h.p.i.) with a 4× objective. Lower panel: All cells identified by the Gen5 algorithm are highlighted in yellow, whereas rounded cells are highlighted in purple. (**b**) Time-dependent changes in the proportion of rounded BHK-21 cells following infection with VSV-GP at the indicated MOIs. Images were acquired at regular 1 h intervals with a 4× objective and analyzed using Gen5. As the total cell number varied over time due to cell proliferation but also virus-induced cytopathic effects, the proportion of rounded cells relative to the absolute cell number was determined. Each point represents the mean of eight wells ± SD. Lines represent the result of a non-linear kinetic fit with global lower asymptote for all curves. Representative data of one out of four experiments, independently performed by different operators, are shown.

Despite significant advancements in automation of assay readouts [9], traditional endpoint titration methods still suffer from limited precision and/or low sample throughput. To overcome this analytical bottleneck, we sought ways to replace the existing readout methods, typically based on the detection of cell lysis, with an assay setup benefiting from the detection of more subtle and earlier virus-induced cellular changes. Furthermore, we implemented a kinetic readout scheme with acquisition of label-free brightfield images over regular time intervals. The cumulative analysis of time-dependent changes increases the precision and accuracy for each sampled well and alleviates the need to incubate cells until a quantifiable infection endpoint is reached. As a model virus for assay development, we used VSV-GP. VSV-GP is an engineered oncolytic virus, based on vesicular stomatitis virus (VSV), in which the original glycoprotein was replaced by the glycoprotein (GP) of lymphocytic choriomeningitis virus (LCMV) [17]. Due to their robust growth characteristics and high susceptibility to productive VSV-GP infection, BHK-21 cells were chosen as the model host cells. Cells were infected at different multiplicities of infection (MOIs) with VSV-GP and imaged at regular time intervals using an automated imaging plate reader. To increase precision by covering as many cells as possible, images were typically acquired using a 4× objective. Higher magnifications were only applied to better visualize the morphological changes determined by the automated software algorithm. Among the measured cellular parameters, the increase over time in the proportion of rounded cells demonstrated the strongest dose dependency and greatest signal-to-noise ratio (Figure 1 and Figure S1a). In each of our analyses, the proportion of rounded cells in relation to the absolute cell number was determined to compensate for time-dependent changes in cell number arising from proliferation and/or virus-induced cytopathic effects. The baseline of rounded cells, which is shared by uninfected cells (mock) and infected cells prior to onset of infection, depends on the definition of a rounded cell. For our assay, cells in which the ratio of the smallest cell diameter to the largest cell diameter exceeded 0.3 were classified as rounded. Lower or higher cutoff values only resulted in all curves being shifted parallel towards higher or lower overall percentages of rounded cells, respectively. However, this might be a useful optimization parameter when testing other cell–virus combinations. VSV-GP infection induced a rapid and dose-dependent increase in the proportion of rounded cells that continued until a common upper asymptote was reached for all MOIs. This progression of virus-induced cell rounding was observed independently of the software algorithm (Gen5 or Columbus) used for image analysis (Figure 1b and Figure S1b). Furthermore, it resembles the changes in cellular fractions determined by a linear classifier, which integrates multiple parameters such as roundness, intensity, area, and surface texture (Figure S1b). This suggests that cell rounding is not only an easily measurable parameter but also representative for the multitude of cellular changes occurring upon infection. A slight increase in rounded cells was also observed in mock infected wells. This is likely a consequence of growing cell density and not critical, as its extent and time-dependent progression never reached the levels of infection-induced cell rounding.

**Figure 2:**
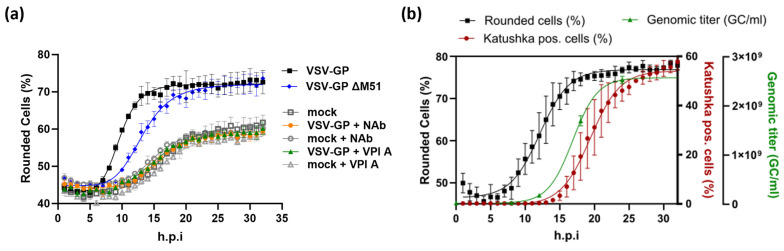
Cell rounding is specific to viral entry and gene expression. (**a**) Cell rounding kinetics determined in BHK-21 cells infected with VSV-GP or a variant thereof with a mutated M protein (VSV-GP ΔM51) at an MOI of 1. Where indicated, viral entry was inhibited by treatment with a neutralizing antibody (NAb; KL25) (10 µg/mL) or viral RNA transcription was inhibited by treatment with the polymerase inhibitor Vesiculopolin A (VPI A) (7.5 µM). Each point represents the mean of three wells ± SD. The same experiment was also performed with infections at MOIs of 10 and 0.1. Results confirmed the findings for MOI 1, which is shown here as a representative experiment. (**b**) BHK-21 cells were infected at an MOI of 0.32 with a VSV-GP variant expressing the red fluorescent Katushka protein. Cell rounding was determined in bright-field images. In the same field of view, fluorescence was determined with an excitation/emission wavelength of 584 ± 20 nm/625 ± 20 nm. Rounded cells and Katushka-positive cells are both expressed as a percentage of the total number of cells in the bright-field image. Each point represents the mean of three wells. A second plate was infected and incubated in parallel and used to harvest supernatants at 0, 8, 12, 18, and 24 h.p.i. Genomic virus titers were determined by qRT-PCR to monitor viral replication and are expressed as genomic copies (GC)/mL. A non-linear 4-parameter logistic model was applied to fit the curve. All curves are shown ± SD.

To confirm the specificity of the observed morphological alterations for VSV-GP infection, cell rounding was tested under conditions in which viral replication was inhibited at different stages. In the presence of a neutralizing antibody, which binds to the viral glycoprotein (GP) and inhibits viral entry, no VSV-GP-dependent rounding of BHK-21 cells could be observed (Figure 2a). Similarly, inhibition of viral RNA transcription and, concomitantly, viral protein expression by the polymerase inhibitor Vesiculopolin A [18] fully prevented virus-induced cell rounding (Figure 2a). The delayed rounding kinetics in cells infected with the genetically modified VSV-GP ΔM51 mutation are consistent with previous reports [19] showing that M protein functions contribute to cell rounding (Figure 2a). The ΔM51 mutation reduces the association of the viral M protein with nuclear core complexes and its ability to inhibit nucleocytoplasmic transport, which might be related to M-protein-mediated cytotoxicity [20,21,22]. The connection between the progression of viral gene expression and cell rounding was further assessed in cells infected with a VSV-GP variant encoding the red fluorescent Katushka protein. The increase in the proportion of Katushka-positive cells was detectable ~7 h after the onset of cell rounding. However, the dependence of cell rounding on viral gene expression shown above (Figure 2a), together with the finding that the exponential viral growth phase preceded the detectability of Katushka (Figure 2b), strongly suggests that this is rather a sensitivity issue than the actual sequence of biological events. Importantly, cell rounding and Katushka expression, which reflects viral protein production, show similar curve progressions (Figure 2b), independent of the MOI used for infection (Figure S2a). While at a higher MOI all kinetics shift towards earlier timepoints, the temporal relation between cell rounding, the emergence of Katushka-positive cells, and the release of progeny virions remains unaffected (Figure S2b). Altogether, these findings show that entry and viral gene expression are a prerequisite for virus-induced cell rounding, which reflects viral growth kinetics in infected cells.

### 2.2. Titer Determination with the Kinetic Infectious Virus Titer (KIT) Assay

**Figure 3:**
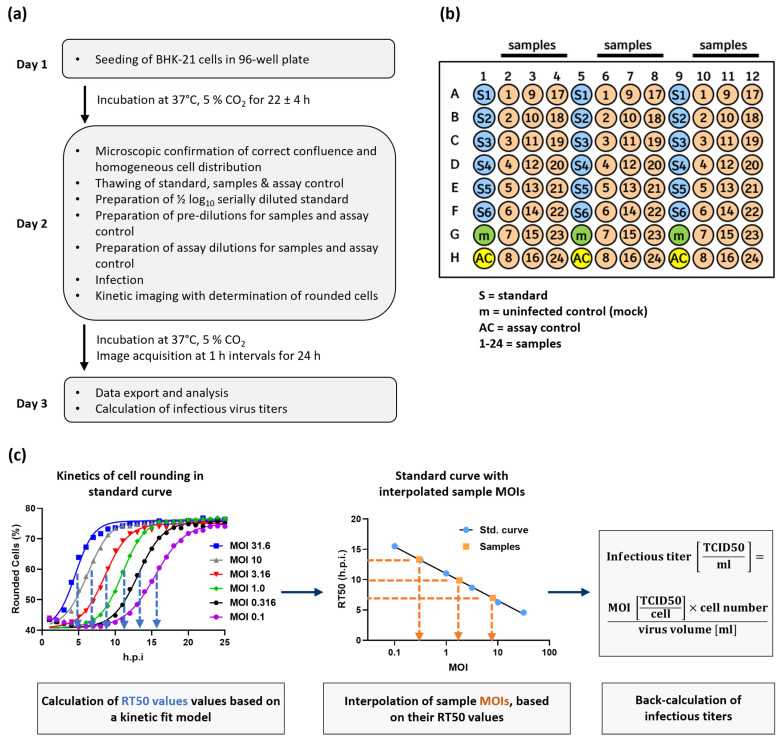
Workflow for performing the kinetic infectious virus titer (KIT) assay. (**a**) Experimental outline for the determination of infectious virus titers using the KIT assay. (**b**) Exemplary plate layout for the KIT assay. The plate contains, in alternating orientation, triplicates of (1) six wells infected with a reference standard virus at ½-log_10_ serially diluted MOIs, (2) uninfected control wells (mock), (3) an assay control which is included on each assay plate and serves for trending purposes of critical assay performance parameters, and (4) up to 24 samples. (**c**) Data analysis workflow to calculate infectious virus titers based on kinetics of infection-induced cell rounding. Further details are given in the text and in the Material and Methods section.

The specificity for viral replication, its strong dose and time dependency, and the simple measurement based on label-free imaging qualified cell rounding kinetics as an attractive readout parameter for our development of an improved setup of infectivity assays. To optimize the method precision and robustness while maintaining sufficient experimental flexibility for routine use of the assay, we employed a Design of Experiment (DoE) approach [23,24]. The factors chosen to be optimized were cell seeding density, media composition, cell pre-culture time, incubation time between cell seeding and infection, and the working range (details and results will be published elsewhere). The setup of the resulting kinetic infectious virus titer (KIT) assay is outlined in Figure 3. Briefly, BHK-21 cells, which are maintained in Glasgow’s MEM (GMEM) supplemented with 6.1% fetal calf serum (FCS) and 4.3% tryptose phosphate broth (TPB), are seeded into 96-well plates at a density of 18,000 cells per well 22 ± 4 h before infection.

To achieve high intermediate precision and accuracy over time, titers are determined in a relative manner, normalized to a reference standard which is included on each plate. The reference standard consists of well-characterized and representative virus material, which is aliquoted and stored under conditions maintaining stability over a long period of time. In our setup, the standard curve consists of six MOIs with ½ log_10_ increments, resulting in a 2.5 log_10_ assay range (MOI 31.6–MOI 0.1). Although dose-dependent differences in the progression of cell rounding were also observed for higher and lower MOIs (Figure 1b), limiting the assay range to the described MOIs with homogeneous rounding kinetics resulted in the highest assay precision. Obviously, this assay parameter should be optimized for other virus–cell combinations. 

When infecting the cells, each sample is applied with a single dose, maximizing the throughput. As, of course, the exact titer of the sample is unknown, the applied dose, i.e., the sample volume resulting in the intended target MOI, is estimated based on previous knowledge about the sample type or production process. The intended target MOI is 1.78, the middle of the working range of the assay, to allow valid measurements within the working range of the assay even if the assumed sample titer differs ±1 log_10_ from the actual titer. If the sample titer cannot be estimated with sufficient certainty, different dilutions of the sample can be applied to ensure that at least one of them falls within the assay working range. Diluting the samples to a fixed target MOI would require pipetting specific volumes for each of the samples. However, the Material and Methods section provides a detailed solution, which helps to avoid this error-prone procedure and allows automated sample preparation. 

Finally, 100 µL of the serially diluted standard as well as the assay dilutions of the samples and the assay control are transferred to triplicate wells of the 96-well plate containing the cells seeded at 100 µL per well on the previous day. Wells in which 100 µL assay medium are added are included as an uninfected control (mock). Even though consistent results were generated irrespective of the positioning on the plate, applying standards and samples in alternating order (Figure 3b) further helps to reduce the possible influence of plate effects, also called plate non-uniformity. Immediately after infection, incubation of the plate with imaging of all wells at regular 1 h intervals using an automated imaging reader is started, with a total duration of 24 h.

For every timepoint of image acquisition, the proportion of rounded cells was determined for each well. Plotting rounded cells (%) against time post-infection (h.p.i.) results in sigmoidal curves, which can be fitted by a model describing an initial lag-phase followed by an exponential increase in the proportion of rounded cells, which culminates in an upper asymptote (Figure 3c) (see Materials and Methods section for details about the model). The upper asymptote reflects a stable condition in which all susceptible cells are affected by virus-induced cytopathic effects and most of them meet the classification threshold qualifying them as rounded. Based on the fit, an RT50 value is determined for each curve. The RT50 value describes the time post-infection at which the half-maximal extent of cell rounding is achieved (inflection point between lower and upper asymptotes of the sigmoidal curves; see blue dotted lines in left panel of Figure 3c). A standard curve can be generated by plotting the six RT50 values of the reference standard dilution series against their respective MOIs. On a semi-log scale, the standard curve is described by a simple linear regression model. Based on the RT50 values of the samples, their MOIs can be interpolated from the standard curve (see orange dotted lines in the middle panel of Figure 3c). The interpolated MOI can then be converted into the original infectious virus titer of the sample (see right panel of Figure 3c). As the KIT assay is a relative method, the determined infectious titers of the samples will always be expressed in the same unit as results from the assay used to characterize the reference standard.

With the described setup of the KIT assay, infectious titers of up to 24 samples can be determined on a single 96-well plate. In our routine use of the assay, the turnaround time for completion of a full plate is less than 45 min, and results are available already 24 h after infection.

### 2.3. Performance Characteristics of the KIT Assay

**Figure 4:**
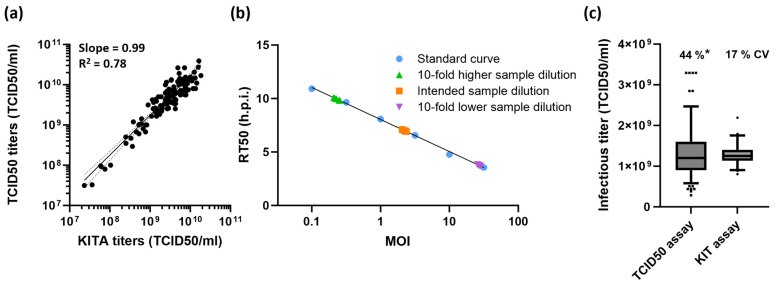
Performance characteristics of the KIT assay. (**a**) Correlation between titers determined with the kinetic infectious virus titer (KIT) assay and the TCID50 assay. A total of 116 VSV-GP samples were analyzed with both assays. For the KIT assay, one determination (triplicate wells) was performed for each sample. TCID50 titers show the mean value of three determinations per sample. For non-linear regression analysis, the sum of squares of the distances of the points from the curve was minimized by weighting by 1/Y^2^. The dotted lines represent the 95% confidence interval (CI) of the curve (slope 0.93–1.13). (**b**) Intra-plate precision of the KIT assay. The KIT assay was used to determine the titer of a sample with an actual titer of 1.39 × 10^9^ TCID50/mL. To control assay performance throughout its working range, the sample was tested at three different target MOIs, which was achieved by three different 10-fold pre-dilutions. For each target MOI, six determinations were performed. Refer to Table S1 for the tabulated results of the experiment. Representative results from one out of five experiments independently performed by two different operators is shown. (**c**) Comparison of TCID50 and KIT assay variability. Box plots show the median value as well as the first (lower end of the box) and third quartiles (upper end of the box), containing 50% of all data points. Whiskers indicate the 2.5%/97.5% percentile. Titers from 269 single determinations are shown for the TCID50 assay. Titers from 90 determinations, representing all values generated in five independent assays described in Figure 4b, are shown for the KIT assay. Assay variability is indicated as coefficient of variation (CV %). * Variability of the TCID50 assays refers to a single determination and can be reduced by averaging result from multiple measurements.

Infectious virus titers of samples obtained from a formulation development study determined by the KIT assay (three wells per sample) correlate well (slope = 0.99; R^2^ = 0.78) with titers determined by TCID50 assay (results from three independent determinations, i.e., three 96-well plates averaged per sample) (Figure 4a). The precision and accuracy throughout the working range of the KIT assay were tested by decreasing or increasing the intended target MOI (1.78), resulting in a 10-fold higher or lower sample dilution, respectively. Six determinations were performed at each of the three target MOIs. Irrespective of the target MOI, the KIT assay showed intra-plate precision with an average coefficient of variation (CV) of 11%, determined in five independent assays, performed by different operators. With an intermediate precision of 17% CV and an overall recovery of 91%, the applied setup for the KIT assay yielded accurate results throughout the complete working range (Figure 4b,c, Table S1). This shows that with only three wells per sample, the variability of the KIT assay is smaller than what can be achieved with our optimized TCID50 assay [9] (44% CV; with a single determination, i.e., one 96-well plate measured per sample) (Figure 4c). A study in which variations regarding the cell seeding density were simulated further confirmed the robustness of the KIT assay. Assays performed with 10,000, 14,000, or 22,000 cells/well showed similar standard curves as the optimized assay with 18,000 cells/well (Figure S3a). Irrespective of the number of cells seeded, all KIT assays yielded sample titers that are comparable to the titers determined by TCID50 assay, but with higher precision (Figure S3b).

**Figure 5:**
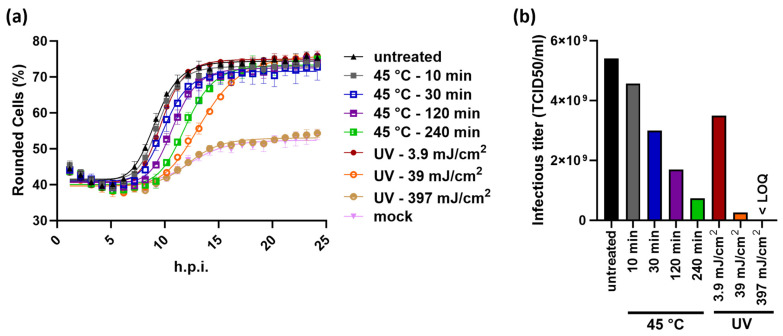
The KIT assay is stability-indicating. (**a**) Time-dependent rounding of BHK-21 cells infected with VSV-GP, subjected to the indicated stress conditions. Following the standard procedure of the KIT assay, all samples were measured in triplicate wells, shown ±SD. Equal sample volumes were used for all infections, which correspond to an MOI of 1.78 for the untreated sample. (**b**) The KIT assay workflow was used to determine infectious virus titers of the samples shown in (**a**) relative to a reference standard. At the highest dose of UV irradiation, no virus-dependent cell rounding occurred (curve indistinguishable from mock), and the infectious virus titer was thus below limit of quantitation (LOQ). Representative results from one out of three independently performed experiments are shown.

The KIT assay was also used to measure VSV-GP subjected to increased temperature or UV irradiation. The forced sample degradation manifested itself in a delayed rounding of infected BHK-21 cells (Figure 5a). This reflects a loss of infectious virus titers, the extent of which correlates with the applied stress level (Figure 5b). Comparative measurements using the TCID50 assay confirmed that the stress-induced loss of infectivity is equally detected by both assays (Figure S4). These findings confirm that the KIT assay is a stability-indicating method, which can also resolve small differences in virus infectivity.

### 2.4. The Principle of the KIT Assay Can Be Applied to Different Virus–Cell Combinations

**Figure 6:**
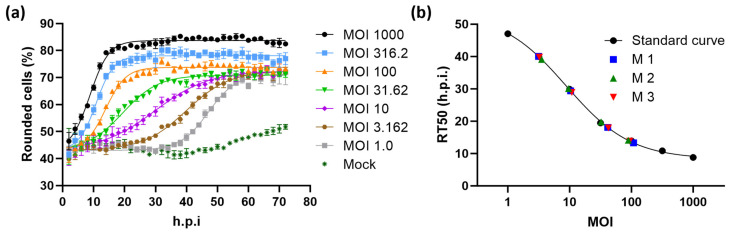
Application of the KIT assay to determine infectious titers of Newcastle disease virus (NDV). (**a**) Time-dependent cell rounding after infection of DF-1 chicken fibroblasts with NDV at the indicated MOI. Each point represents the mean of three wells. (**b**) RT50 values of the infections shown in (**a**). A sigmoidal four-parameter logistic fit was applied to the RT50 values, plotted against the respective MOI, to generate a standard curve. RT50 values and the interpolated MOIs of three samples (M 1–M 3) tested at four different concentrations are depicted on the standard curve. Refer to Table S2 for the tabulated results of the experiment. Representative results from one out of two independently performed experiments are shown.

Morphological changes in host cells are a common phenomenon upon infection with various types of viruses. This suggests that the principle of measuring the kinetics of cell rounding to determine infectious titers is not limited to VSV-GP. To confirm this, we performed a feasibility study applying the principle of the KIT assay to determine titers of Newcastle disease virus (NDV) based on rounding of DF-1 chicken fibroblasts [25]. As observed with VSV-GP in BHK-21 cells, NDV infection induced dose- and time-dependent rounding of DF-1 cells (Figure 6a). While all curves started at a common basal value of rounded cells, the height of the upper asymptote depended on the applied MOI. It is not surprising that the relation between virus dose and cell rounding differs from the observations made with VSV-GP. Factors contributing to these characteristics include the apparently less effective spread and induction of cell death by NDV, which allows a certain level of proliferation of regular shaped DF-1 cells and limits the proportion of rounded cells, especially at lower MOIs. These virus and host cell dependencies are reflected by the RT50 values, which were determined using our kinetic curve fit model and exhibited a sigmoidal curve shape when plotted against the respective MOIs (Figure 6b, Table S2). The precision and accuracy of the measurements for NDV were evaluated by interpolating the MOIs of three NDV samples (M 1–M 3) at four different concentrations. The mean CV of 13% and recovery of 107% of the actual sample titer highlight that the KIT assay not only allows precise and accurate determination of VSV-GP titers but can also be applied to completely unrelated virus–cell combinations, for which titers can be determined with the same high throughput.

To further corroborate its broad applicability, the KIT assay principle was also applied to HeLa cells infected with parapoxvirus ovis (ORFV). As shown before, infection resulted in cell rounding with dose-dependent kinetics (Figure S5a). The different curve progressions did not allow to apply the same fit model as for the previous viruses. Therefore, we used area under the curve (AUC) analyses as a simple and convenient alternative, which resulted in a sigmoidal standard curve when plotted against the respective MOI (Figure S5b). This shows that although slight adaptations regarding the data analysis might be required, kinetic imaging provides a simple and broadly applicable tool to determine viral infectivity. 

## 3. Discussion

**Figure 7:**
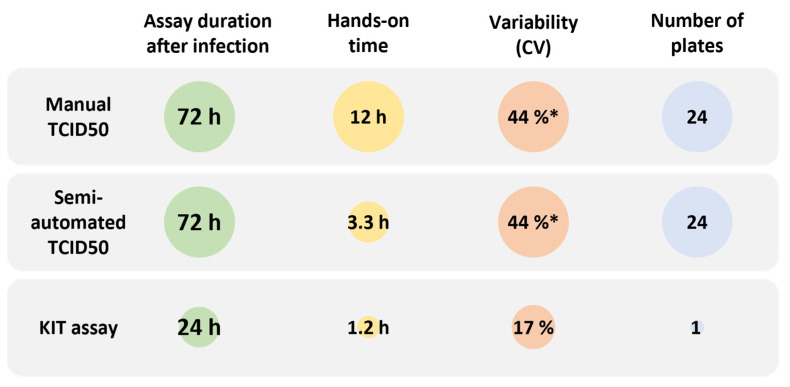
Comparison of performance characteristics of different infectivity assays. The hands-on time and number of processed multi-well plates represent values for the measurement of 24 samples. * Variability of the TCID50 assay refers to a single determination and can be reduced by averaging results from multiple determinations.

Infectivity is the most critical quality attribute for many virus-based therapeutics because it determines their ability to enter target cells, deliver their therapeutic payload, and/or replicate. Therefore, the ability to monitor this fundamental mode of action is key for evaluating the potency, safety, and efficacy of virus-based therapeutics. The pharmaceutical industry thus has a strong interest in infectivity assays that allow precise determination of infectious virus titers in a fast and cost-efficient manner. However, traditional assay principles, such as the TCID50 or plaque assay, suffer from high variability and limited sample throughput. This creates an analytical bottleneck, especially for complex process and formulation development studies. Here, precise measurements of large sample numbers are required to make informed decisions, which allow, for example, to maximize production yields and therefore decrease production costs. To overcome these limitations, we developed a high-throughput method to determine infectious virus titers based on automatic quantification of infection-induced cell rounding in kinetically acquired bright-field images. The kinetic infectious virus titer (KIT) assay (Figure 3) is characterized by its straightforward principle, low running costs, short time to results, and superior precision as well as a ~10-fold increased throughput compared to the traditional TCID50 assay (Figure 7).

The TCID50 assay has been widely utilized in various virological disciplines for many years due to its simplicity and the independence of special instrumentation. However, as an endpoint dilution assay, it requires every sample to be diluted to a point at which infection is absent in the inoculated culture wells or caused by only a single (or few) infectious virus particle. Furthermore, the assay design includes infection in multiple replicates for each dilution step to calculate the TCID50, describing the sample dilution at which 50% of the inoculated wells show signs of infection. This high number of pipetting steps and the laborious readout procedure, which often relies on manual microscopic evaluation of infection-induced cytopathic effects (CPEs), limit the sample throughput. Furthermore, the need to wait until a single initial infection event results in an infection outcome, which by manual microscopic evaluation can be unambiguously identified as such, prolongs the time to result. We have previously shown that automation of the sample dilution procedure combined with automated image-based CPE evaluation reduces the hands-on time approximately fourfold [9]. Automated image analysis can, for both plaque and TCID50 assays, be combined with virus-specific immune staining. The resulting increased sensitivity allows for an earlier measurement with operator-independent results [10,26]. However, this comes at the cost of additional working steps and makes the assay dependent on a constant supply with a critical, possibly expensive, reagent. In our KIT assay, we enable an early readout based on bright-field images without additional staining steps. To do so, a software algorithm quantifies infection-induced changes in the percentage of rounded cells. Cell rounding occurs early after infection with a variety of viruses and is likely the result of virus-induced subversion of the actin cytoskeleton [27] and/or represents the onset of CPEs. Our data show that infection-induced cell rounding occurs already within a few hours after infection and depends on viral entry as well as viral gene expression (Figure 1 and Figure 2). The delayed cell rounding kinetics observed for the VSV-GP ΔM51 variant emphasize the involvement of M protein functions in this process and are in alignment with previous reports showing that mutations disrupting the expression of the alternative translation products M2 and M3 (M33,51A) reduce rounding of infected BHK-21 cells [19]. The specificity of cell rounding for viral infection is further confirmed by the time-dependent progression, which strongly correlates with the applied virus dose.

The algorithm-based image analysis allows for reliable assessment of subtle infection-induced cellular changes, much earlier than possible by eye. Implementation of this readout procedure could therefore also be an option for traditional infectivity assays, where the presence/absence of CPEs or the number of plaques is determined at a single timepoint. Several examples of using artificial intelligence (AI) to detect CPEs in bright-field images have been presented previously [13,28,29]. However, it is often not fully understood on which properties the AI bases its decision. Using these sophisticated software tools in infectivity assays therefore entails the challenges of robust validation, quality control, and regulatory compliance, especially when applying the assay under GMP conditions. This is more straightforward if simple and transparent readout parameters, such as cell rounding, are used. This requires, of course, that the virus used induces a specific change in the respective host cell that can be measured in an automated manner in living cells without additional staining steps.

In addition to its user-friendliness, the major benefit of the KIT assay lies within the principle of kinetic measurements. The curve fit of the time-dependent progression of cell rounding takes into consideration the values obtained over the complete assay duration. Thus, a curve parameter such as the RT50 has a much higher accuracy and precision in comparison to a measurement at a single timepoint. This makes the measurement robust and less sensitive to outliers and yields precise results, with a CV < 20% using only three wells per sample (Figure 4). Our TCID50 assay, which depends on CPE evaluation at a single timepoint, exhibits a CV of about 45% with a single determination per sample. This is already quite good for such a traditional infectivity assay, where a variability of ±0.5 log_10_ is not uncommon [7,8]. However, to reach the precision of the KIT assay, data from six determinations, equaling six 96-well plates, would have to be averaged (Figure 7) [9]. Of note, we successfully upscaled the KIT assay to 384-well plates without major adaptations. If higher assay precision is required in the KIT assay, this could therefore be achieved by increasing the number of replicates, with only a minor reduction in sample throughput. Due to the kinetic measurement, it is also not necessary to define a fixed timepoint for the readout as images can simply be acquired until all curves reach the upper asymptote, represented by the stable percentage of rounded cells. This results in a broad assay range and in most cases abolishes the need to test samples at different concentrations, which has a positive effect on the number of samples which can be measured in parallel. As prolonged measurements negatively affect the time to results, a reasonable and practical assay range and thus duration for image acquisition should be chosen. Altogether, the presented design of the KIT assay allows to measure up to 24 samples on a single 96-well plate within 24 h, while keeping the variability below a CV of 20%.

As the kinetics of cell rounding reflect productive viral replication, the KIT assay is able to measure differences not only in concentration but also in viral functionality. The KIT assay is thus a stability-indicating method (Figure 5). Due to the determination of sample titers relative to a reference standard, the influence of day-to-day variability, which is often caused by cultured cells, is minimized, making the KIT assay a powerful tool for long-term stability studies. Moreover, it is expected that the collection of kinetic data enables the KIT assay to resolve differences in viral fitness, which are reflected by altered infection and replication kinetics. These differences can, for example, be caused by viral mutations and might be neglected by traditional infectivity assays, which depend on a single endpoint readout.

The power of kinetically acquired data is also appreciated in studies using the xCELLigence Real-Time Cell Analysis (RTCA) system for the determination of infectious virus titers [14]. However, due to the need for using specialized plates, measuring cellular impedance causes relatively high running costs, and the instrument is provided by a single manufacturer. In contrast, automated imaging readers are commonly available and can be combined with either commercially available or custom-built automated plate incubators. Importantly, independent algorithms with different ways of evaluating cell rounding yielded highly comparable quantifications of the proportion of rounded cells (Figure S1), showing that this readout method does not depend on a special feature of a specific software. The flexibility regarding the used instrumentation and software is especially advantageous if the assay needs to be aligned between multiple sites with different pre-existing lab configurations, as is often the case in the pharmaceutical industry.

We used our engineered oncolytic virus VSV-GP as a model for the initial assay development. However, it is likely that the KIT assay can also be applied to, and might thus be of interest for, a growing number of other VSV-based therapeutics or vaccines [30,31,32,33,34]. Importantly, we showed that the KIT assay also allows for determining infectious titers of unrelated virus types, such as Newcastle disease virus (NDV) and parapoxvirus ovis (ORFV) (Figure 6 and Figure S5). This is not surprising as similar morphological changes are commonly observed [35], indicating that the principle of assessing the kinetics of infection-induced cell rounding is applicable for a large proportion of CPE-inducing viruses. However, the biological characteristics of the virus replicating in a specific host cell, including cellular growth rates, effectiveness of viral spread, and the strength of infection-induced cytopathicity, will certainly require adaptations regarding experimental details or data analysis. For certain virus–cell combinations, cell rounding might not even be the ideal readout parameter. With the wide range of conventional or AI-based image analysis tools, it should, however, be possible to find a suitable infection-specific morphological readout parameter with strong time and dose dependency for most types of viruses. This makes the kinetic infectivity assay a versatile tool and therefore also attractive for academic research, where, in most cases, a high level of flexibility is required. Furthermore, the additional level of information regarding virus kinetics might be a bigger advantage for academic research in comparison to the pharmaceutical industry, where functional differences or genetic variants will routinely be identified by orthogonal analytics to determine, for example, the proportion of infectious particles or genomic integrity.

## 4. Materials and Methods

### 4.1. Cell Lines

BHK-21 cells (CLS, London, UK, #603126) were cultured in Glasgow’s MEM (GMEM) (Gibco, Waltham, MA, USA, #21710) supplemented with 6.1% fetal calf serum and 4.3% tryptose phosphate broth (Thermo Fisher, Waltham, MA, USA, #18050039). The baseline of BHK-21 cells classified as rounded already at the beginning of the assay might increase if the cell passage is too high. While the overall performance of the assay is still good, this effect reduces the dynamic range as the baseline shifts closer to the upper asymptote of rounded cells that is reached upon infection. To avoid this, we used BHK-21 cells only up to passage 20 for the KIT assay. DF-1 cells (Elabscience, Houston, TX, USA, #CL-0279) were cultured in DMEM (Gibco #11960) supplemented with 2% Ultroser G (Sartorius, Göttingen, Germany), 4 mM L-glutamine, and 1 mM sodium pyruvate. HeLa cells were cultured in MEM with GlutaMAX^TM^ supplement (Thermo Fisher #41090036), supplemented with 9.1% fetal calf serum. Cells were passaged 2–3 times per week before reaching full confluence. For passaging, cells were washed with PBS and detached by incubation with TrypLE^TM^ Select Enzyme (Thermo Fisher #12563011) for 6–8 min. Cells were taken up in medium, counted using a NucleoCounter NC-200 (ChemoMetec, Allerod, Denmark), and then further cultivated or seeded for an assay. Cells were incubated at 37 °C under 5% CO_2_ in a humidified atmosphere. Sterility and absence of mycoplasma were confirmed for all used cell lines.

### 4.2. Kinetic Infectious Virus Titer (KIT) Assay

**Sample dilution and infection procedure**

BHK-21 cells were seeded into 96-well flat bottom plates (Thermo Fisher #161093) at a density of 18,000 cells in 100 µL 22 ± 4 h before infection. All pipetting steps for cell seeding and virus dilutions were either performed manually or in a semi-automated fashion using Integra pipetting robots (Mini96, VIAFLO96, Assist Plus). All dilutions for the reference standard, samples, and assay control were prepared using supplemented cell culture medium as a diluent. 

In our typical assay setup for VSV-GP, the reference standard curve consists of six MOIs with ½ log_10_ increments, resulting in a 2.5 log_10_ assay range (e.g., MOI 31.6–MOI 0.1). To broaden the assay range, lower MOIs can be included in the reference standard curve, but the duration of image acquisition might have to be prolonged to ensure that all curves reach the constant upper asymptote of rounded cells.

In addition to the reference standard, an assay control (optional) can be included. The assay control is a representative virus sample, which is included on every assay plate. It is treated just like all other samples, but the infectious virus titers determined for the assay control should be documented together with other critical assay details (e.g., operator, cell passage, cell pre-culture time, time between seeding and infection, and batch ID of critical reagents) to monitor assay performance over time. As for all other samples, measurement of the assay control is performed at a single concentration, i.e., the MOI. In our assays, the target MOI used for infection was 1.78, which lies in the middle of the standard curve and allows valid measurements within the working range of the assay even if the assumed sample titer differs ±1 log_10_ from the actual titer. MOI calculations are made based on expected titers, which are informed by previous knowledge about the sample type or production process. One challenge that comes with a constant target MOI is that different sample volumes would have to be used for infection for each sample, which is error-prone and represents a considerable limitation for assay automation. To bypass this issue, expected sample titers are rounded to the next integer log_10_ value and only then used to calculate the sample volumes required to reach the target MOI (refer to Table 1 for an example of possible dilution schemes). Serial 10-fold sample dilutions are used to obtain the required sample concentration in volumes that are easy to pipette. Due to the previous rounding step, the volumes of pure sample required to infect at an MOI of 1.78 always differ by a factor of 10 between samples with different expected titers. Thus, only the number of 10-fold pre-dilutions must be adapted, but all pipetted volumes remain constant. This is also true for the final dilution step (in our example: dilution of 192 µL pre-diluted virus in 408 µL medium), in which the highest 10-fold pre-dilution is used to prepare the assay dilution, which is used to infect the BHK-21 cells. 

Finally, 100 µL of the serially diluted standard as well as the assay dilutions of samples and the assay control were transferred to replicate wells (triplicates in the standard assay setup) of the 96-well plate containing the cells seeded on the previous day. Wells in which 100 µL assay medium (no virus) was added served as the uninfected control (mock), and 100 µL medium was added to unused wells to maintain consistent culturing conditions throughout the whole plate.

The overall infection procedure was the same when titers of Newcastle disease virus (NDV) were determined in DF-1 cells or when cell rounding of HeLa cells infected with ORFV was determined. The only differences were the seeding density of 15,000 cells/well for DF-1 cells and 8000 cells/well for HeLa cells and the different MOIs used for infection. In the case of VSV-GP and NDV, the MOI was defined as TCID50 per cell, whereas in the case of ORFV, the MOI was defined as plaque-forming units (PFUs) per cell, depending on the infectivity assay used to initially characterize the samples.

**Automated image acquisition**

Immediately after infection, incubation of the plate with imaging of all wells at regular intervals (1 h for VSV-GP and ORFV, 2 h for NDV) using an automated Cytation5 (Agilent/BioTek, Santa Clara, CA, USA) multimode imaging reader in combination with an automated BioSpa (Agilent/BioTek) incubator was started. Values of the very first measurement (0 h.p.i.) were excluded from all analyses as fogged-up lids, occurring due to initial temperature differences, did not allow reliable automated analysis of the wells. The total duration of image acquisition depended on the virus, the MOIs used for infection, and the specific purpose of the experiment. Imaging was performed using a wide field of view objective with 4× magnification and laser autofocus. Expression of the red fluorescent Katushka protein was monitored at an excitation/emission wavelength of 584 ± 20 nm/625 ± 20 nm.

**Image analysis**

Analysis of cell rounding and other infection-induced morphological changes was performed on bright-field images. Unless stated otherwise, image analysis was performed using Gen5 (Agilent/BioTek). Cellular analysis in Gen5 was restricted to an area with 3000 µm diameter in the center of the image, avoiding the outmost areas of the well with possible lower optical quality. Within the total number of identified cells (object size: 5–75 µm), the software determined the rounded cell proportion. Cells are analyzed by approximating their shape with an ellipse and defined as rounded if the ratio of the smallest diameter to the largest diameter exceeds 0.3. A different threshold value would shift the absolute value of % rounded cells of the upper and lower asymptotes. Importantly, however, the time-dependent progression of cell rounding would be maintained, and due to the calculation of the titers relative to a reference standard, this would not significantly affect the results. Of note, the common upper asymptote of rounded cells reached by most MOIs after a sufficient time of viral replication and spread remained below 100%, irrespective of the algorithm used for quantification. This suggests either that some cells are not susceptible to infection and do not undergo cell rounding, or that not all cells round up entirely and remain below the threshold for being classified as rounded.

Columbus^TM^ (PerkinElmer, Shelton, CT, USA) was used as a complementary software tool to determine cell rounding and other cellular changes occurring upon infection. Two approaches with different levels of complexity were applied. First, as conducted by the Gen5 software (version 3.14), cells were defined as rounded if the ratio of the smallest diameter to the largest diameter exceeded 0.3. Secondly, a linear classifier model based on the Columbus^TM^ PhenoLOGIC^TM^ technology was applied to discriminate elongated versus rounded cells. The linear classifier was trained with both cell populations (around 200 cells each across many different images) and then all images were analyzed. For the classification of cells, Columbus integrated different parameters including roundness, width/length ratio, cell area, intensity, and cell surface texture.

**Statistical analysis**

In the KIT assay, the outcome of the image analysis is the percentage of rounded cells in each well at a specific point of time after infection. If these two factors are plotted against each other with time post-infection on the x-axis and % rounded cells on the y-axis, this results in a sigmoidal curve (Figure 3). The following kinetic curve fit model is used to determine the RT50 value, i.e., the time after infection at which the half-maximal cell rounding is reached, from each of the sigmoidal curves. The model was adapted from formulas describing bacterial growth kinetics, which show similar curve progression [36].

$$y=LA\;\frac{UA-LA}{1+10^{-k\left(x-RT50\right)}}$$

where *LA* = lower asymptote, the value of which is dependent on the baseline of rounded cells per well; *UA* = upper asymptote, the value of which is the maximum % rounded cells that is reached based on the defined image analysis parameters; *k* = slope at the inflection point of the curve; and *RT*50 = time after infection at which the half-maximal extent of cell rounding is reached, which is identical to the inflection point of the curve. 

The RT50 values of the different doses of the reference standard were plotted against the corresponding known MOIs, resulting in a standard curve (Figure 3). The heterogeneous curve progressions for the rounding of ORFV-infected HeLa cells did not allow to apply the same fit model as for VSV-GP or NDV. Thus, area under the curve (AUC) analyses were applied as a convenient alternative. For VSV-GP in BHK-21 cells, the standard curve is reflected by a semi-log line, whereas a sigmoidal four-parameter logistic fit model was applied for NDV in DF-1 cells and ORFV in HeLa cells. Based on the determined RT50 or AUC values of the assay control and the samples with unknown titers, their MOIs can be interpolated from this standard curve. The interpolated MOIs, together with the known number of cells seeded per well and the volume of the sample that was added per well, are then used to calculate the infectious titer. The calculated infectious virus titer has the same unit in which the infectivity of the reference standard sample is expressed.

**Working range of the KIT assay**

The working range of the KIT assay is strongly dependent on the biological interplay between the tested virus and the type of host cell, which determines assay characteristics such as the speed of viral replication and spread as well as the kinetics of the morphological changes upon infection. Thus, a suitable MOI range needs to be identified for each virus–cell combination. The lower the MOI is, the longer it takes until a quantifiable extent of infection-induced cell rounding is reached. To enable a readout early after infection, which constitutes a great advantage of the KIT assay as compared to traditional infectivity assays, and to avoid well-to-well variability caused by an overly low number of infection events per well, it is recommended to work at a high enough MOI range. This, however, makes it mandatory to exclude matrix effects during assay development, which might have a negative influence on the performance or specificity of the assay. When accepting a longer assay duration (e.g., 40 h) and potentially higher variability at low MOIs, VSV-GP samples could still be measured at an MOI of 0.0032 (Figure 1b). Further assuming that a 1:20 dilution of the sample is sufficient to exclude matrix effects, the minimal required sample titer which could still be measured in the KIT assay was ~1 × 10^4^ TCID50/mL. The KIT assay is therefore a powerful method for high-throughput measurements of samples with medium to high titer, rather than a method suitable to measure residual levels of infectious virus in potentially cytotoxic matrices.

### 4.3. TCID50 Assay

The semi-automated version of the TCID50 assay with algorithm-based evaluation of cytopathic effects (CPEs) was performed as described previously [9]. BHK-21 cells were seeded in 96-well plates. After 24 h, adherent cells were infected with 8 replicates of a ½-log_10_ serial sample dilution. At ~72 h post-infection (h.p.i.), bright-field images were acquired using a Cytation5 Multimode Imaging Reader (Agilent) with a 4× objective. An algorithm determined the cell area in each well. Wells with a cell area below a certain threshold were classified as CPE-positive. The model of Spearman/Kärber or Reed/Münch [4,5] was used to calculate the infectious virus titer. 

### 4.4. qRT-PCR

TaqMan^TM^ qRT-PCR was used to monitor VSV-GP replication in infected BHK-21 cells. Genomic viral RNA was isolated from cell culture supernatants using the KingFisher Flex System (Thermo Fisher) in combination with the MagMAX^TM^ Viral/Pathogen Nucleic Acid Isolation Kit (Applied Biosystems, Waltham, MA, USA, #A42352). Reverse transcription of RNA and cDNA amplification were conducted in single-tube format using the TaqMan^TM^ Fast Virus 1-Step Master Mix (Applied Biosystems #4444432). A VSV-GP-specific TaqMan probe and primers binding the *m* and *gp* genes were used. Signals were quantified relative to a synthetic RNA standard, and the results are expressed as genomic copies (GC) per mL.

## Figures and Tables

**Table 1:** Examples for rounding of expected sample titers, dilutions, and volumes to prepare the assay dilutions. * Volumes given for the final assay dilution result in a total volume of 600 µL. As only triplicate infections with 100 µL each are performed per sample, this gives enough spare volume for losses during pipetting.

Sample ID	Expected Titer (TCID50/mL)	Rounded Titer (TCID50/mL)	Target MOI	Volume of Undiluted Sample per Well (µL)	Number of 10^−1^ Sample Pre-Dilutions	Volume Pre-Diluted Sample (µL) *	Volume Medium (µL) *
Sample 1	2.72 × 10^8^	1 × 10^8^	1.78	0.32	2	192	408
Sample 2	8.16 × 10^8^	1 × 10^9^	1.78	0.032	3	192	408
Sample 3	5.09 × 10^9^	1 × 10^10^	1.78	0.0032	4	192	408

## Data Availability

The raw data supporting the conclusions of this article will be made available by the authors on request.

## Supplementary Materials

The following supporting information can be downloaded at: https://www.mdpi.com/article/10.3390/ijms25158076/s1.
