# Supplementary material for: High-Throughput Determination of Infectious Virus Titers by Kinetic Measurement of Infection-Induced Changes in Cell Morphology
Source: Int J Mol Sci. 2024 Jul 24;25(15):8076. doi: 10.3390/ijms25158076 (PMC11311753; doi:10.3390/ijms25158076)
Supplement: Supplementary file 1 [file ijms-25-08076-s001.zip › ijms-3065963-supplementary.pdf]

International Journal of
Molecular Sciences

MDPI

Research Article

High throughput determination of infectious virus titers by kinetic measurement of infection-induced changes in cell morphology

Dominik Hotter [1], Marco Kunzelmann [2], Franziska Kiefer [1], Chiara Leukhardt [1], Carolin Fackler [1], Stefan Jäger [3] and Johannes Solzin [1]

[1] Boehringer Ingelheim Pharma GmbH & Co. KG, Viral Therapeutics Center, Birkendorfer Straße 65, 88397 Biberach an der Riß, Germany
[2] Boehringer Ingelheim Pharma GmbH & Co KG, Development Biologicals, Birkendorfer Straße 65, 88397 Biberach an der Riß, Germany
[3] Boehringer Ingelheim Pharma GmbH & Co KG, Central Nervous System Diseases Research, Birkendorfer Straße 65, 88397 Biberach an der Riß, Germany
* Correspondence: johannes.solzin@boehringer-ingelheim.com

Supplements

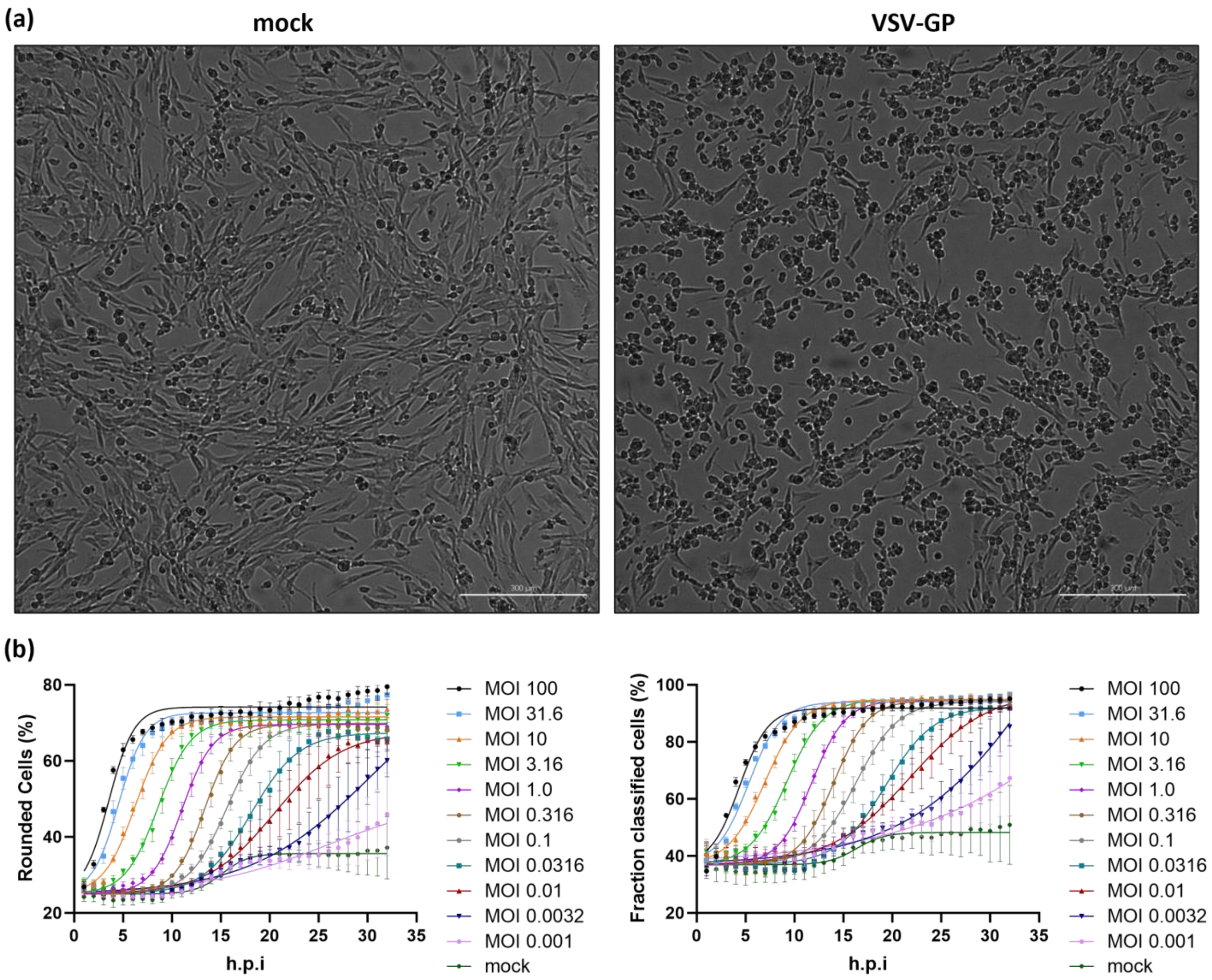

Figure S1. Different algorithms yield similar results for infection-induced cellular changes. (a) Representative bright-field images of BHK-21 cells, which were left uninfected (mock) or infected with VSV-GP at a MOI of 1, acquired at 10 hours post-infection using a 10x objective. Scale bar: 300 µm. **(b)** BHK-21 cells were infected with VSV-GP at the indicated MOI. Bright field images of the infected cells were acquired in 1 h intervals with a 4x objective and analyzed applying different algorithms of the Columbus image analysis tool. To confirm that the determination of rounded cells is comparable throughout independent algorithms, images of the same experiment were analyzed in this figure and Figure 1b. Each point represents the mean of eight wells ± SD. Lines represent the result of a non-linear kinetic fit with global lower asymptote for all curves. Left panel: The proportion of rounded cells normalized to the total cell number was determined. Cells were defined as rounded if the ratio of their smallest cell diameter to the largest cell diameter exceeds 0.3 (compare to results presented in Figure 1 generated using Gen5). Right panel: Two cell fractions were discriminated based on a linear classifier, which integrates multiple cellular parameters including roundness, length-width-ratio, area, intensity and surface texture.

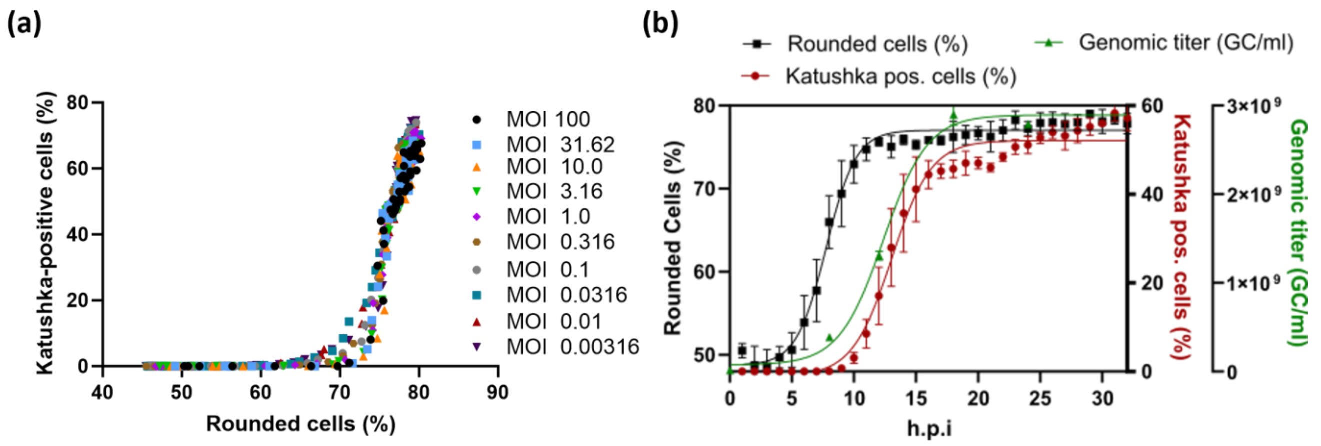

Figure S2. Temporal relation between cell rounding, viral gene expression and viral replication. (**a**) BHK-21 cells were infected at the indicated MOI with a VSV-GP variant expressing the red fluorescent Katushka protein. The proportion of rounded cells relative to the total cell number was determined based on bright-field images. In the same field of view, fluorescence was determined with an excitation/emission wavelength of 584±20 nm/625±20 nm. Katushka positive cells are expressed as percentage of the total number of cells in the bright field image. Each point represents the mean of four wells. The graph shows the relation between the proportion of rounded cells and Katushka-expressing cells, whereas time is not accounted for. (**b**) BHK-21 cells were infected at an MOI of 10 and brightfield and fluorescence images were acquired as described for (a). Rounded cells and Katushka-positive cells are both expressed as percentage of the total number of cells in the bright field image. Each point represents the mean of three wells. A second plate was infected and incubated in parallel and used to harvest supernatants at 0, 8, 12, 18 and 24 h.p.i. Genomic virus titers expressed as genomic copies (GC) per ml were determined by qRT-PCR to monitor viral replication. A non-linear 4 parameter logistic model was applied to fit the curve. All curves are shown ± SD.

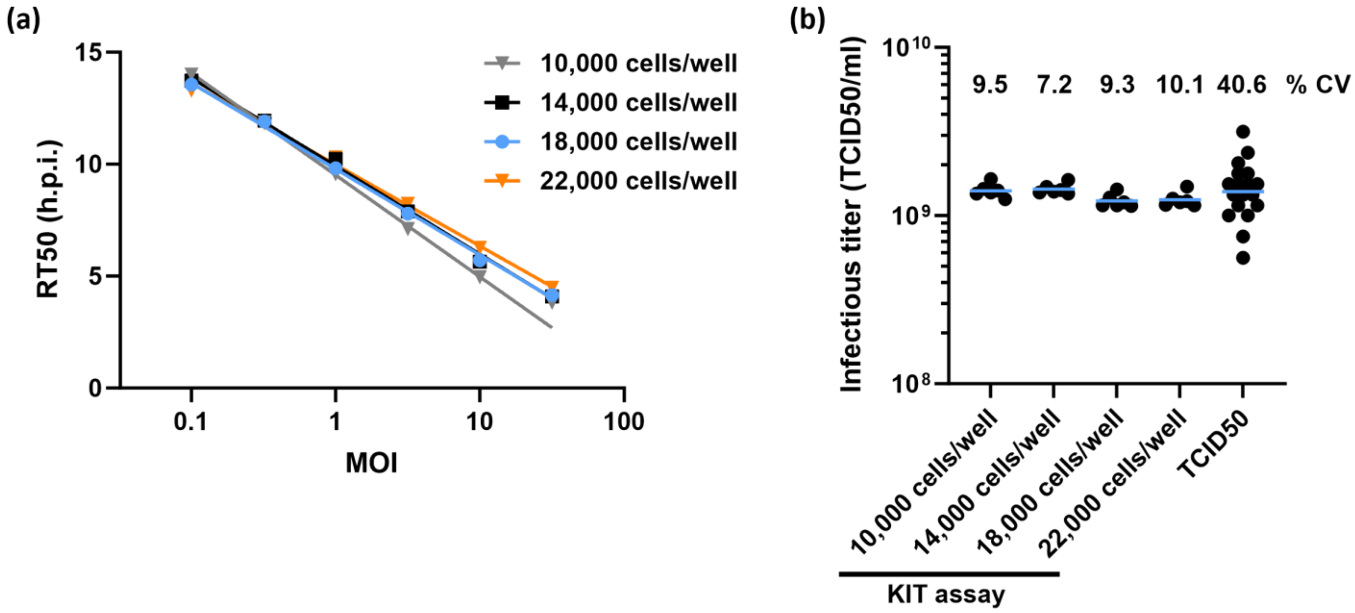

Figure S3. The KIT assay is robust against variations in cell seeding density. (a) BHK-21 cells were seeded in 96-well plates at the indicated cell seeding density. 24 h after seeding, cells were infected in triplicate wells with VSV-GP using the indicated MOI. Based on the kinetic progression of cell rounding, RT50 values were determined for each condition and plotted against the respective MOI to generate a reference standard curve for each cell seeding density. **(b)** Seeding and infections were performed as described in (a) to determine the infectious virus titer of the same sample in six determinations for every cell seeding density in the KIT assay. For comparison, infectious virus titer of

the same material was determined in 12 determinations using the TCID50 assay. The blue lines indicate the geometric mean of the individual measurements. The variability of the multiple determinations is indicated as the coefficient of variation (% CV).

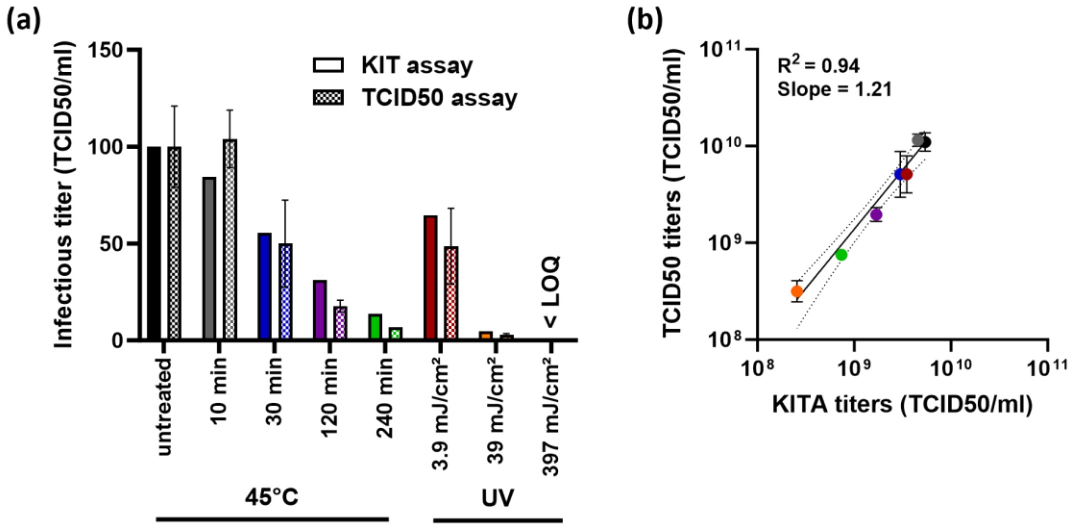

Figure S4. Stress-induced loss of infectivity is equally detected by KIT and TCID50 assay. (a) VSV-GP was subjected to different levels of temperature- or UV-stress. Infectious virus titers of the stressed samples were determined by KIT (also refer to Figure 5) and TCID50 assay. Following the standard procedure for the KIT assay, each sample was measured in triplicate wells. The titers determined by TCID50 assay represent the mean ± SD of three determinations. **(b)** Correlation between KIT and TCID50 titers determined as described in (a). For non-linear regression analysis, the sum-of-squares of the distances of the points from the curve was minimized by weighting by $1/Y^2$. The dotted lines represent the 95% confidence interval (CI) of the curve (slope 1.01 – 1.39). Representative results of one out of two independently performed experiments are shown.

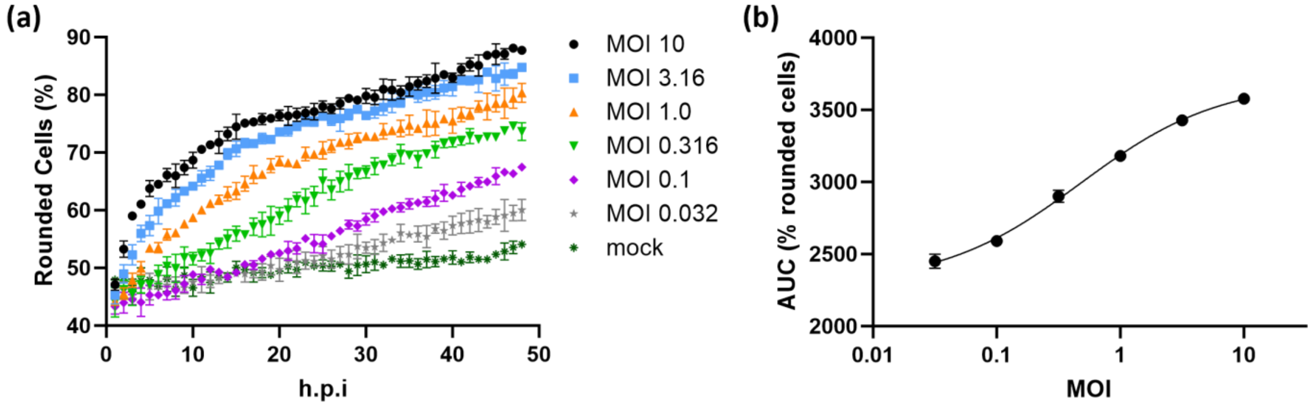

Figure S5. Application of the KIT assay principle to determine cell rounding induced by parapoxvirus ovis (ORFV). (a) Time-dependent cell rounding after infection of HeLa cells with ORFV at the indicated MOI. Each point represents the mean of three wells ± SD. **(b)** Area under the curve (AUC) values calculated for the infections shown in (a). A sigmoidal four parameter logistic fit was applied to the RT50 values plotted against the respective MOI, to generate a standard curve. Mean values of three wells ± SD are shown. Representative results of one out of two independently performed experiments are shown.

Table S1. Tabulated results of intra-plate precision of the KIT assay. The KIT assay was used to determine the titer of a sample with an actual titer of 1.39E+09 TCID50/ml. To control assay performance throughout its working range, the sample was tested at three different target MOIs, which

was achieved by three different 10-fold pre-dilutions. For each target MOI, six determinations were performed (see Figure 4b). Variability is expressed as coefficient of variation (CV).

Target MOI	Measured titer (TCID50/ml)	Mean titer (TCID50/ml)	CV (%)	Overall CV (%)	Overall mean titer (TCID50/ml)	Overall mean recovery (%)
17.8	1.50E+09	1.52E+09	4.10	10.86	1.35E+09	97.02
	1.47E+09					
	1.57E+09					
	1.53E+09					
	1.59E+09					
	1.43E+09					
1.78	1.15E+09	1.24E+09	5.61			
	1.25E+09					
	1.28E+09					
	1.25E+09					
	1.33E+09					
	1.16E+09					
0.18	1.28E+09	1.29E+09	8.10			
	1.44E+09					
	1.40E+09					
	1.17E+09					
	1.21E+09					
	1.26E+09					

Table S2. Tabulated results of precision and accuracy of the KIT assay for determination of NDV titers. Time-dependent cell rounding after infection of DF-1 chicken fibroblasts with NDV was determined at different MOIs. RT50 values were calculated and plotted against the corresponding MOI. A sigmoidal four parameter logistic fit was applied to the RT50 values to generate a standard curve, which was used to interpolate MOIs of three samples (M 1-M 3) tested at four different concentrations (see Figure 6b). Recovery of the actual sample titer (3.80E+08 TCID50/ml) and variability expressed as coefficient of variation (CV) are shown.

	Measured titer (TCID50/ml)							
Target MOI	M 1	M 2	M 3	Mean titer (TCID50/ml)	CV%	Overall mean titer (TCID50/ml)	Overall CV%	Recovery (%)
3.16	3.76E+08	4.24E+08	3.88E+08	3.96E+08	6.36	4.05E+08	12.92	106.64
10	3.94E+08	3.65E+08	4.13E+08	3.91E+08	6.11			
31.6	4.97E+08	3.90E+08	5.11E+08	4.66E+08	14.22			
100	4.10E+08	3.38E+08	3.56E+08	3.68E+08	10.27			
